# The Role of Artificial Intelligence in Addressing Menopausal Mental Health Challenges

**DOI:** 10.5935/1518-0557.20260019

**Published:** 2026

**Authors:** Rowaida Sadat, Koray Gorkem Sacinti, Andrea Panattoni, Stefano Luisi

**Affiliations:** 1 Department of Obstetrics and Gynecology, Ankara University School of Medicine, Ankara, Turkey; 2 Department of Obstetrics, Gynecology and Reproductive Sciences, Yale School of Medicine, New Haven, CT, USA; 3 Division of Assisted Reproductive Medicine and Infertility, Division of Obstetrics and Gynecology, Maternal and Child Health Unit, Pisa University Hospital (AOUP), Pisa, Italy; 4 Division of Obstetrics and Gynecology, Department of Clinical and Experimental Medicine, University of Pisa, Pisa, Italy

**Keywords:** menopause, mental health, artificial intelligence, AI-driven solutions

## Abstract

Menopause marks a crucial transition in a woman’s life and is often accompanied by physical and psychological changes that can adversely affect mental health. Depression, anxiety, cognitive changes, and sleep disturbances are common during the menopausal transition, yet they are frequently underdiagnosed and undertreated, particularly in lowand middle-income countries. Emerging technologies, especially artificial intelligence (AI), offer new opportunities to narrow this care gap. Although AI has shown considerable promise in identifying menopause-related physical health conditions (e.g., osteoporosis and endometrial cancer), its use for mental health during this life stage remains limited. We discuss the potential of AI-driven tools-including machine learning algorithms, digital therapeutics, symptom trackers, and large language models-to improve the detection, monitoring, and personalized management of menopause-associated mental health disorders. By integrating genetic, clinical, lifestyle, and wearable data, AI systems may help predict risk, identify symptom patterns, and support tailored interventions. These approaches could enable scalable, accessible, and cost-effective mental healthcare, reduce stigma, and address service gaps. Harnessing AI in this area offers a significant opportunity to improve quality of life for millions of women worldwide.

Menopause is a major life stage, typically beginning in the mid-40s and extending over several years (Delanerolle et al., 2025); the mean age at the final menstrual period is approximately 50 years (Riecher-Rössler, 2020). This transition is often accompanied by physical and psychological changes that can affect mental health and overall quality of life. Menopause-related mental health conditions-including depression, anxiety, cognitive decline, and sleep problems-are often underdiagnosed or poorly managed (Riecher-Rössler, 2020).

Declining estrogen levels during the menopausal transition have been linked to neuroendocrine dysregulation, which may contribute to the onset or worsening of affective symptoms, including mood disturbances, irritability, and depressive episodes. This period also frequently overlaps with other life challenges-such as aging, changes in social roles, and societal expectations-which can further exacerbate mental health concerns (Riecher-Rössler, 2020). Despite the burden of these symptoms and their long-term implications, menopause care remains inadequate in many settings, particularly in lowand middle-income countries (Delanerolle *et al*., 2025). Cultural and societal barriers may also deter women from seeking care, complicating the management of menopause-related mental health problems. As the global population ages, it has been estimated that by 2030 more than 1.2 billion women worldwide will be experiencing or will have already experienced menopause (Delanerolle *et al*., 2025).

The complexity of menopausal symptoms-spanning both physical and mental health-calls for innovative approaches to diagnosis and management. Artificial intelligence (AI) in mental healthcare could help address challenges in the availability, acceptability, and accessibility of services (Nilsen *et al*., 2022). Machine learning (ML) and deep learning (DL) techniques have shown promise in diagnosing and managing menopause-related health concerns. For example, AI has been applied to osteoporosis detection, where algorithms can help identify women at risk and support earlier, more tailored treatment (Cruz *et al*., 2018). DL models have demonstrated encouraging diagnostic performance for osteoporosis, with reported sensitivities of 81%-91% (Cruz *et al*., 2018). Similarly, ML approaches have been used to detect hot flashes, with sensitivities up to 87% and specificities up to 97% (Cruz *et al*., 2018). AI models have also been applied to screening for endometrial cancer in postmenopausal women, with reported sensitivity of 86% and specificity of 83% (Pergialiotis *et al*., 2018). These examples underscore AI’s potential to improve diagnostic precision and enable more personalized care for women during menopause.

Despite these advances in menopause-related physical health, the use of AI to address mental health during this life stage remains underexplored. This is a major opportunity: AI could enhance identification, diagnosis, and management of menopause-associated mental health disorders. AI may also support broader post-reproductive health management by assessing comorbidities, estimating long-term health risks, tracking symptoms, and aiding treatment decisions. By analyzing genetic, lifestyle, and clinical data, AI systems can help predict individual risk profiles and identify symptom patterns over time. In addition, data from wearables and symptom-tracking apps (e.g., heart rate, sleep, mood, and activity) can provide real-time insight into health status and potential symptom triggers. These capabilities can support more personalized care plans, including lifestyle interventions, cognitive behavioral therapy, and other evidence-based strategies. Digital therapeutics, symptom trackers, and large language models may also improve access to support for depression, anxiety, and cognitive symptoms, while enabling continuous monitoring and timely feedback for both women and clinicians.

A key advantage of AI-enabled approaches is the potential to provide scalable and affordable support. In settings where mental health professionals are scarce, AI-driven platforms may offer continuous assistance for symptom management and emotional well-being. This could reduce pressure on healthcare systems while improving timely access to care. Moreover, AI tools may help reduce stigma around menopause and mental health by enabling women to seek support privately and without fear of judgment.
